# Simple colorimetric monitoring of chlorogenic acid in coffee washing water using Ag-modified faujasite zeolite

**DOI:** 10.1007/s10661-026-15430-x

**Published:** 2026-05-13

**Authors:** João Otávio Donizette Malafatti, Elaine Cristina Paris, Luiz Henrique Capparelli Mattoso

**Affiliations:** National Nanotechnology Laboratory for Agriculture (LNNA), Embrapa Instrumentação, XV de Novembro St., 1452, São Carlos, SP 13560-970 Brazil

**Keywords:** Chlorogenic acid, Silver-modified faujasite, Zeolite-based materials, Colorimetric sensing, Environmental monitoring, Coffee water

## Abstract

Silver-based materials are widely recognized for their optical, electronic, and chemical properties, which make them attractive for sensing applications. Among these materials, silver-modified zeolites stand out due to their high surface area, chemical and dispersion stability, and the possibility of tailoring surface interactions to improve affinity toward specific analytes. In this study, a faujasite zeolite framework modified with silver ions (AgFAU) is investigated for colorimetric sensing of chlorogenic acid (CGA). The detection was carried out using a digital image colorimetry (DIC) approach, in which red, green, and blue (RGB) parameters were used for the quantitative optical analysis of colored dispersions. Based on the DIC analysis, CGA could be quantitatively monitored in the concentration range of 1–20 mg L^−1^, with a limit of detection in the range of 0.47–1.17 mg L^−1^ (average, 0.8 ± 0.3 mg L^−1^), consistent with values reported for similar colorimetric sensors. The proposed approach was successfully applied to the detection of CGA in coffee wastewater. In addition, the system showed good specificity toward structurally related polyphenols and high reproducibility (RSD ≤ 6%), demonstrating its suitability as a simple and easy-to-interpret strategy for CGA monitoring.

**Supplementary information:**

The online version contains supplementary material available at 10.1007/s10661-026-15430-x.

## Introduction

Chlorogenic acid (CGA), scientifically known as 5-caffeoylquinic acid (5-CQA), is a polyphenolic compound widely metabolized by plants such as coffee, grapes, citrus fruits, tomatoes, and potatoes (Kundu & Vadassery, 2019). CGA is considered a valuable compound in the food, pharmaceutical, and cosmetic industries because of its antioxidant, anti-inflammatory, antimicrobial, and antitumor properties (Munteanu & Apetrei, 2021, 2022; Pimpley et al., 2020). Coffee is one of the most widely consumed beverages worldwide and represents a commodity of high economic relevance. During coffee processing, the beans undergo washing steps to remove impurities, followed by drying and skin removal. Although these washing stages improve the quality of the final product, they also promote the release of soluble coffee compounds into aqueous effluents (Das, 2021). Given its high abundance and chemical reactivity, CGA has been proposed as an indicator of total polyphenolic content and product quality (Santos et al., 2011). Therefore, from an environmental perspective, the control and monitoring of coffee washing wastewater are essential.

The monitoring of CGA molecules in aqueous systems has attracted increasing interest, particularly in environmental and agro-industrial processes. CGA can be detected and quantified using several analytical techniques, including fluorescence spectroscopy (Hu et al., 2023; Liu et al., 2021), electrochemical methods (Abbas & Amin, 2022; Awan et al., 2020; Ganesamurthi et al., 2022), liquid chromatography (Santanatoglia et al., 2024; Soto et al., 2022), ultraviolet-visible (UV-vis) spectrophotometry (Wang et al., 2019), and near-infrared (NIR) spectroscopy (Navarra et al., 2017). Although these methods offer high sensitivity and reliable performance, they frequently require laborious sample preparation and complex instrumentation. As a result, current technological developments have focused on simpler and more portable analytical strategies, aiming to reduce cost, enable miniaturization, and facilitate in situ analysis.

Colorimetric sensing approaches represent a promising alternative for chemical detection, as the interaction between the analyte and the sensing array induces a visible color change, enabling simple and direct visual monitoring (Jia et al., 2021; Lan et al., 2019; Piriya V.S et al., 2017). This rapid color variation results from changes in light absorption behavior as a function of analyte concentration (Lan et al., 2017; Popa et al., 2020; Xu et al., 2019). In such systems, sensing molecules can be arranged or chemically modified within arrays to enhance their affinity toward specific target compounds, allowing the detection of a wide range of analytes, including ions (Amin et al., 2022; Irfan et al., 2022; Sengan et al., 2020), dyes (Xiao-wei et al., 2018), pesticides (Qian & Lin, 2015), proteins (Mao et al., 2016), and DNA (Xu et al., 2019). In addition, smartphone-assisted colorimetric approaches have been explored as practical tools for data acquisition and processing (Jiang et al., 2024).

Silver nanoparticles (AgNPs) exhibit localized surface plasmon resonance in the visible region through the interaction of conduction electrons with electromagnetic radiation. Interactions with target molecules can modify this optical response, resulting in color changes that enable colorimetric detection (Fiorati et al., 2020; Prosposito et al., 2020; Teodoro et al., 2019). Accordingly, silver-based materials have been widely explored as sensing platforms for interaction with different classes of analytes, including pesticides (Hoang et al., 2021), ions (Rossi et al., 2021), and drugs/pharmaceutical compounds (Iqbal et al., 2025; Scroccarello et al., 2019; Shaban et al., 2021). Despite these advantages, a major challenge associated with AgNPs is their limited colloidal stability in aqueous suspensions, which often results in aggregation processes (Bélteky et al., 2019). The use of scaffolds and support materials has been proposed to improve dispersion homogeneity, providing their stabilization. In this context, zeolites are crystalline aluminosilicates composed of tetrahedrally coordinated aluminum (Al^3^⁺) and silicon (Si^4^⁺) atoms arranged in a three-dimensional framework with well-defined cavities and high porosity. Their high structural stability and surface reactivity arise from the presence of labile charge-balancing cations (Cheng et al., 2019; Reinoso et al., 2018). Among zeolite families, faujasite (FAU) is particularly attractive, as it presents one of the largest pore openings among common zeolite frameworks, favoring the interaction of molecules from the external environment with accessible active sites (Paris et al., 2020). Consequently, FAU exhibits suitable features for anchoring Ag⁺ ions through the cation-exchange of Na⁺ ions, enabling the development of stable silver-modified materials.

Despite the growing interest in chlorogenic acid analysis, colorimetric approaches specifically designed for CGA detection in aqueous and agro-industrial matrices remain limited and insufficiently explored (Yao et al., 2020). The novelty of this work lies in the use of a zeolite-based material combined with smartphone-assisted colorimetric analysis for CGA detection. This strategy allows direct application in complex matrices, such as coffee washing water, without the need for conventional analytical instrumentation. In this context, the present study proposes colorimetric sensing based on faujasite zeolite surface-modified with Ag⁺ ions (AgFAU) for the detection of CGA. The colorimetric response was evaluated using red, green, and blue (RGB) color parameters obtained through digital image analysis supported by a smartphone-based application. The selectivity of the AgFAU system was investigated in the presence of potential interferents, including structurally related polyphenols such as caffeic acid, quinic acid, and catechin, as well as in coffee bean washing water. Overall, this work demonstrates the feasibility of an alternative and simple colorimetric approach for CGA monitoring, highlighting its potential application in quality control and environmental assessment within coffee production processes.

## Materials and methods

### Materials

Silica (Tixosil 333) was kindly donated by Rhodia Solvay. Sodium aluminate, silver nitrate (AgNO_3_), chlorogenic acid (CGA), caffeic acid (CFA), quinic acid (QA), and catechin (CAT) were purchased from Sigma-Aldrich. Sodium hydroxide, sodium chloride, sodium citrate, citric acid, sodium carbonate, and sodium bicarbonate were purchased from Synth. Dried *Coffea arabica* beans were kindly donated by C. A. Vasconcelos, located in São Sebastião da Grama, São Paulo, Brazil.

### FAU synthesis

Faujasite (FAU) zeolite was synthesized following the method reported by (Meirelles et al., 2023). Briefly, 3.75 g of sodium aluminate was dissolved in 50 mL of distilled water and subsequently mixed with 50 mL of sodium hydroxide solution containing 10.8 g of NaOH (Synth) under magnetic stirring at room temperature. After homogenization, silica (16 g) was gradually added until the formation of a homogeneous gel. The synthesis gel was estimated as 8.2 Na_2_O/1.0 Al_2_O_3_/13.7 SiO_2_/285 H_2_O. The resulting gel was statically aged for 24 h, promoting the organization of aluminosilicate species and the initial nucleation process before crystallization. The gel was then subjected to hydrothermal treatment at 100 °C for 2 h under magnetic stirring to improve the homogeneity of the reaction medium. Finally, the white precipitate was recovered by centrifugation, washed with distilled water until the supernatant reached pH 8, and dried in an oven at 80 °C.

### FAU-Ag^+^ ion exchange (AgFAU)

The ion-exchange procedure for replacing sodium (Na⁺) with silver (Ag⁺) cations on the FAU zeolite surface was carried out based on the high lability of the charge-balancing ions in the zeolite framework. FAU (1 g), previously dried in an oven at 80 °C for 24 h, was deagglomerated and dispersed in 50 mL of deionized water using a probe ultrasonicator for 1 min at 10% amplitude. Subsequently, this suspension was mixed with 0.16 g of AgNO_3_ previously dissolved in 50 mL of deionized water. The resulting mixture was homogenized under magnetic stirring for 24 h. The silver-exchanged zeolite (AgFAU) was then recovered by centrifugation at 8000 rpm for 10 min at 25 °C and dried in a circulating oven at 80 °C for 24 h.

### Characterizations

X-ray diffraction (XRD) analyses were performed using a Shimadzu diffractometer (model LabX XRD-6000), employing Cu–Kα radiation (*λ* = 1.5406 Å), with 2θ ranging from 5 to 80° and a scanning speed of 1° min^−1^. Field-emission scanning electron microscopy (SEM) and energy-dispersive spectroscopy (EDS) analyses were carried out using a JEOL microscope (model JSM-6510).

Surface area and porosity were determined by N_2_ physisorption/desorption isotherms using a Micromeritics analyzer (model ASAP 2020), and the Brunauer–Emmett–Teller (BET) method was applied for surface area calculation. Zeta potential measurements were performed using a Malvern Instruments Zetasizer (model Nano ZS90) to assess the colloidal stability of the particles.

Fourier-transform infrared spectroscopy (FTIR) was used to evaluate the vibrational modes of functional groups present in the sensor array showing structural changes. The analyses were performed using a Bruker VERTEX FTIR spectrometer, ranging from 4000 to 400 cm^−1^ with 32 scans, and a resolution of 4 cm^−1^.

### Chlorogenic acid (CGA) detection

Colorimetric detection experiments were performed in a total volume of 4 mL, using an AgFAU dispersion (1 mg mL^−1^), with the addition of 200 µL of sodium carbonate/bicarbonate buffer (pH 10.7). Alkaline conditions promote the deprotonation of phenolic hydroxyl groups and the formation of colored species derived from polyphenols, resulting in enhanced optical responses suitable for colorimetric analysis.

The buffer solution was obtained by mixing 45 mL of sodium carbonate solution (0.2 mol L^−1^) with 5 mL of sodium bicarbonate solution (0.2 mol L^−1^). Different concentrations of CGA (1, 2.5, 5, 7.5, 10, 15, and 20 mg L^−1^) were added to the AgFAU dispersion i, and the resulting systems were analyzed by UV-vis spectroscopy and digital image colorimetry (DIC).

RGB color parameters relative to red (R), green (G), and blue (B) were extracted using an RGB color detector smartphone application and used for subsequent data analyses. The CGA samples were labeled as CGA_X, where *X* corresponds to the respective CGA concentration.

Images were acquired using a portable mini photo studio (InstaFold) equipped with cold-white LED illumination (6000 K) and fixed internal dimensions (38 cm height, 36 cm length, and 39 cm depth). The setup included a top opening to accommodate the smartphone, ensuring a fixed working distance and minimizing the influence of external light sources during image acquisition. A Redmi Note 8 smartphone was used for image acquisition in automatic mode. All images were captured under identical conditions, maintaining the same positioning, lighting, and acquisition parameters throughout the experiments. The controlled environment provided by the photo studio minimized variations in exposure and white balance, ensuring reproducibility of the RGB measurements. The RGB values were extracted from the center region of interest for all samples to ensure consistency in the analysis.

#### Digital image data treatment and calibration

The RGB values obtained from each sample were processed separately for the red (R), green (G), and blue (B) channels. For each concentration, measurements were performed in triplicate, and the average values were calculated. A blank sample containing AgFAU in water (without CGA) was used as reference. The RGB values were corrected according to:

1 $$\triangle\mathrm I=I_{sample}-I_{blank}$$

Where *I* represents the intensity of each RGB channel.

Calibration curves were constructed for each channel as a function of CGA concentration. Due to the non-linear behavior observed over the full concentration range, polynomial models were used to describe the analytical response.

#### Limit of detection (LOD) and limit of quantification (LOQ)

The sensitivity of the method was evaluated using a linear region at lower concentrations. The slope obtained from this region was used to calculate the limit of detection (LOD) and limit of quantification (LOQ). The limit of detection (LOD) and limit of quantification (LOQ) were estimated from the RGB color space data according to Eqs. (2) and (3), respectively (Mohamed & Shalaby, 2019):

2 $$LOD=\frac{3{S}_{B}}{m}$$

3 $$LOQ=\frac{10{S}_{B}}{m}$$

where *S*_b_ represents the standard deviation of the blank signal, and *m* is the slope of the calibration curve.

#### Repeatability

Repeatability was evaluated using a fixed CGA concentration (10 mg L^−1^) analyzed in quintuplicate. The relative standard deviation (RSD) was calculated according to Eq. (4):

4 $${RSD}_{n}= \frac{SD (n)}{Mean(n)}$$

where Mean(*n*) represents the average value, and SD(*n*) corresponds to the standard deviation of *n* replicate measurements.

#### Selectivity

The interfering species selected for CGA monitoring included two structurally related polyphenols, caffeic acid (CFA) and quinic acid (QA). In addition, catechin (CAT), a plant-derived polyphenol, and a sodium chloride solution (0.05%, w v^−1^) were evaluated. The interference assays were performed using a concentration of 20 mg L^−1^ for each polyphenolic compound. Tests were carried out for each interfering species individually, both in the absence and in the presence of CGA at a concentration of 20 mg L^−1^.

#### pH effect

The effect of pH on CGA monitoring was evaluated by adjusting the system to pH 6 and pH 3. The assays were performed as described in the “FAU Ag + ion exchange (AgFAU)” section using the maximum analyte concentration (20 mg L^−1^). Citrate buffer solutions at both pH values were prepared using 0.1 mol L^−1^ citric acid solution (A) and 0.1 mol L^−1^ sodium citrate solution (B). For the pH 3 buffer, 46.5 mL of solution A was mixed with 3.5 mL of solution B. For the pH 6 buffer, 9.5 mL of solution A was combined with 41.5 mL of solution B.

### Coffee grain analysis

Wastewater tests were performed by spiking the samples with known CGA concentrations (20, 10, and 1 mg L^−1^) in order to evaluate matrix effects and potential interferences in polyphenol determination. For sample preparation, 100 mg of dried coffee beans was dispersed in 100 mL of water and maintained under agitation for 1 h to simulate the release of soluble compounds under conditions representative of coffee processing effluents. The resulting yellowish suspension was centrifuged at 8000 rpm for 10 min at 25 °C to remove suspended particles.

The influence of sample dilution (10× and 50×) on the colorimetric response was evaluated to identify the most suitable optical conditions. Principal component analysis (PCA) was performed using the RGB color parameters to discriminate the coffee washing water signal and to assess the contribution of CGA within the complex aqueous matrix.

#### Recovery (%)

Recovery experiments were performed using coffee samples containing known amounts of CGA in order to evaluate the applicability of the proposed method in a real matrix. The samples were diluted (50 ×) prior to analysis to minimize matrix effects. Known concentrations (*C*_added_) of CGA (10 and 20 mg L^−1^) were added to the diluted coffee samples, and the resulting systems were analyzed under the same conditions described for the calibration experiments. The RGB values were obtained and processed as previously described. The concentration found (*C*_found_) was calculated from the calibration curve, and the recovery values were determined according to:

5 $$Recovery \,(\%)= \frac{{C}_{found}}{{C}_{added}}$$

All measurements were performed in triplicate, and the average values were used for recovery calculation.

### Data processing

The RGB data were processed and analyzed using polynomial regression models, where each color channel (R, G, and B) was independently fitted to the CGA concentration. The data obtained from the RGB color space parameters were analyzed using principal component analysis (PCA). PCA was performed using the RGB data obtained from independent measurements. Each measurement was treated as an individual observation, and the data were centered prior to analysis to focus on variations associated with CGA concentration.

## Results and discussion

### Physicochemical characterization of AgFAU

**Fig. 1:**
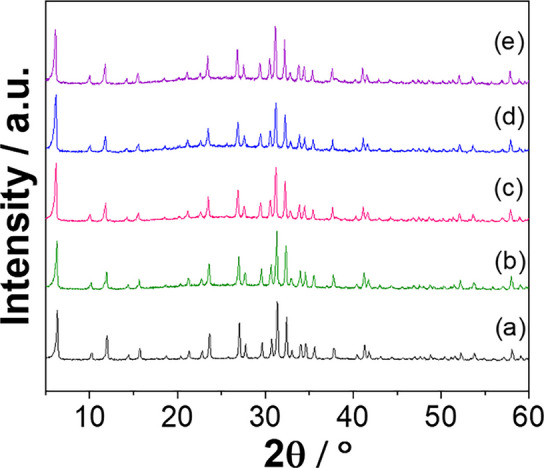
X-ray diffraction (XRD) patterns of AgFAU after (**a**) synthesis, (**b**) exposure to water, (**c**) exposure to buffer solution (pH 10.7), (**d**) exposure to CGA (20 mg L^−1^), and (**e**) exposure to CGA (20 mg L.^−1^) in buffer solution (pH 10.7)

Initially, X-ray diffraction (XRD) was employed to confirm the crystalline structure of the synthesized faujasite zeolite (FAU). The diffraction pattern of the synthesized material (Fig. 1a) is consistent with the characteristic reflections of FAU, according to the JCPDS card no. 43–0168. The diffractograms show the FAU structure is preserved after exposure to aqueous media and to the chlorogenic acid (CGA) polyphenol, even under alkaline conditions (pH 10.7). A slight increase in the background intensity can be observed after exposure to CGA-containing aqueous media or buffer solution (Fig. 1c–e), which may be attributed to the presence of adsorbed organic species, without affecting the characteristic diffraction peaks of the FAU framework. These results demonstrate the high structural stability of the FAU framework under the experimental conditions, which is an important requirement for its application as a sensing material in aqueous suspensions containing CGA.

**Fig. 2:**
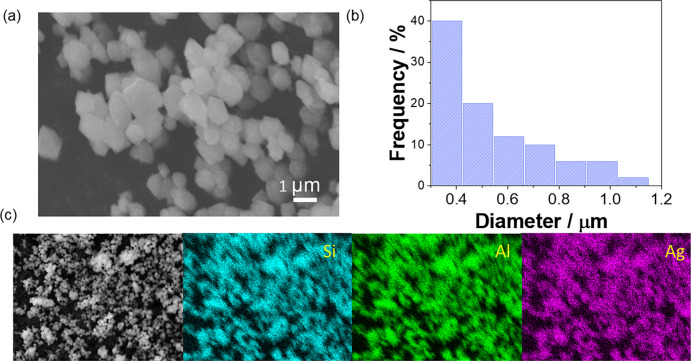
**a** SEM micrograph of AgFAU particles and **b** SEM–EDS elemental mapping of AgFAU obtained from a representative region

The morphological features and elemental composition of FAU were investigated by scanning electron microscopy coupled with energy-dispersive spectroscopy (SEM and SEM–EDS). As shown in Fig. 2, the synthesized zeolite particles exhibit a faceted morphology. The particle size distribution, obtained from the analysis of 50 particles, indicates sizes in the range of 0.5 ± 0.2 µm, displaying a high degree of homogeneity in both size and shape (Fig. 2b).

SEM–EDS analysis was employed to assess the elemental composition of the material, and the presence of silver (Ag) was confirmed in the AgFAU sample, as shown in Fig. 2b. The elemental maps reveal the coexistence of aluminum (Al), silicon (Si), and silver (Ag) signals, indicating a successful loading of silver species associated with the FAU particles. Quantitative EDS analysis showed the presence of O, Na, Al, Si, and Ag, with compositions ranging from 47.1 to 48.7 wt% O, 3.2 to 8.3 wt% Na, 10.4 to 11.9 wt% Al, 18.6 to 21.6 wt% Si, and 11.0 to 19.3 wt% Ag (excluding carbon contribution). On average, the Ag content was approximately 15 wt%, confirming effective silver incorporation into the FAU structure. This behavior is consistent with the well-known cation-exchange capability of zeolitic materials, which arises from the lability of their charge-balancing ions and enables the incorporation of metal species (Paris et al., 2020; Sellaoui et al., 2021).

The surface properties of the zeolite were evaluated by N_2_ physisorption/desorption analysis (Table 1). The synthesized FAU exhibited a high specific surface area of 643 m^2^ g^−1^. After Ag⁺ incorporation, the surface area decreased to 395 m^2^ g^−1^ for AgFAU. This reduction was accompanied by a decrease in the external surface area from 37 to 25 m^2^ g^−1^, as well as a decrease in pore volume from 0.31 to 0.19 cm^3^ g^−1^. These changes indicate that the incorporation of Ag⁺ ions affects both the accessible external surface and the internal porous structure of the zeolite, which is consistent with ion-exchange processes occurring within the FAU framework and partial pore occupation or blockage. Similar behavior has been reported for metal ion incorporation in zeolitic materials (Shichalin et al., 2024). Despite these reductions, significant surface area and porosity are preserved in AgFAU, which is advantageous for the interaction between the sensing material and the analyte in aqueous media.

**Table 1:** Textural properties of FAU and AgFAU determined by N_2_ adsorption–desorption isotherms using the BET method

Sample	Surface area BET (m^2^ g^−1^)	External surface area (m^2^ g^−1^)	Pore size (Å)	Pore volume (cm^3^ g^−1^)
FAU	643	37	19.5	0.31
AgFAU	395	25	19.2	0.19

**Fig. 3:**
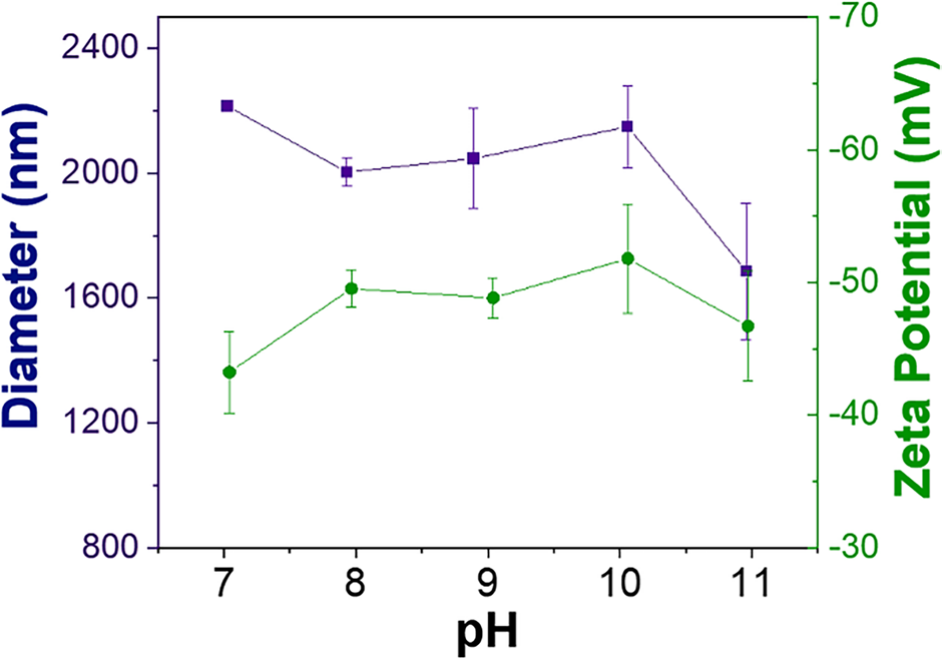
Zeta potential and dynamic light scattering (DLS) analysis (mean ± SD, *n* = 3) of AgFAU suspensions as a function of pH (7–11)

The colloidal stability of AgFAU in aqueous suspension was evaluated by zeta potential measurements and dynamic light scattering (DLS), as shown in Fig. 3. The material exhibited stable behavior over a pH range from 7 to 11, which corresponds to the working conditions employed in the colorimetric experiments. DLS analysis revealed agglomerate sizes in the range of approximately 2000–2200 nm, indicating the formation of particle clusters in suspension.

The larger sizes observed by DLS compared to SEM measurements can be attributed to the different measurement principles of the techniques. While SEM provides the size of individual dried particles, DLS measures the hydrodynamic diameter in suspension, which includes solvation effects and particle aggregation. Considering the primary particle size observed by SEM, these results suggest the formation of small aggregates rather than extensive agglomeration, which is consistent with the observed colloidal stability.

The colloidal stability may be attributed to the high surface charge of the zeolite particles, with zeta potential values around −48 ± 3 mV. Such negatively charged surfaces are typically characteristic of FAU-type zeolites (Awala et al., 2015). In general, colloidal systems with absolute zeta potential values above 20 mV are considered electrostatically stable due to enhanced repulsive interactions between particles (Guo et al., 2018; Jiang et al., 2009; Peres et al., 2023). Overall, these results demonstrate that AgFAU forms stable aqueous suspensions under the experimental conditions, which is essential for obtaining reproducible and reliable optical responses in colorimetric sensing applications.

### Colorimetric detection of CGA using AgFAU

#### CGA standard solutions

**Fig. 4:**
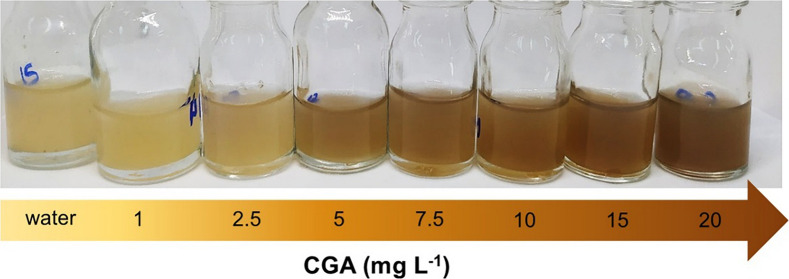
Digital images of AgFAU suspensions containing increasing concentrations of CGA (1–20 mg L^−1^), showing the progressive colorimetric response under controlled illumination conditions

The optical behavior of AgFAU in the presence of CGA at concentrations ranging from 1 to 20 mg L^−1^ (approximately 0–56 µmol L^−1^) is shown in Fig. 4. A gradual color change from yellow to brown is observed as the CGA concentration increases. This variation is consistent with the changes observed in the RGB parameters, which were extracted from digital image colorimetry (Table S1). The concentration-dependent color response of the AgFAU system is consistent with previously reported silver-based colorimetric systems used for chemical detection (Dong et al., 2021; Teodoro et al., 2019). In addition, the colorimetric response allows visual detection at a CGA concentration of 1 mg L^−1^.

**Fig. 5:**
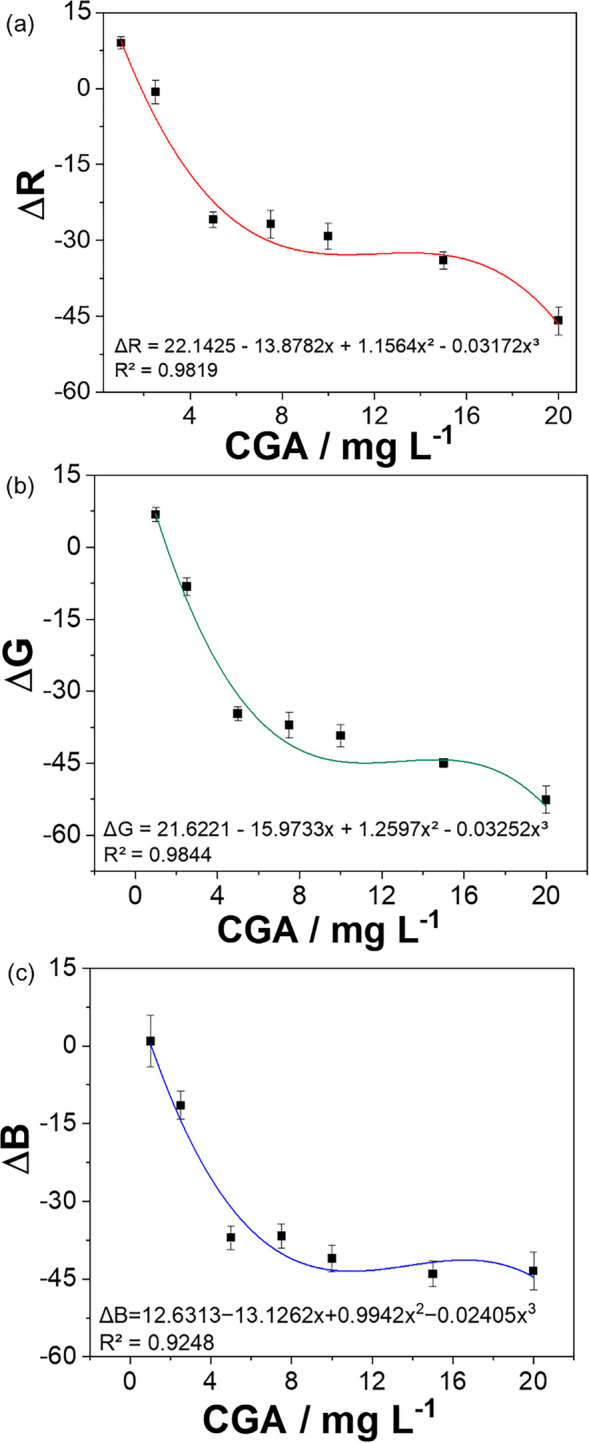
Quantitative determination of CGA using independent polynomial regression models applied to RGB parameters based on red (**a**), green (**b**), and blue (**c**) color space parameters

To enable quantitative analysis, calibration models were developed based on the RGB parameters. The corrected signals (ΔR, ΔG, and ΔB) were calculated by subtracting the corresponding blank values (FAU-water) from each sample as shown in Table 2.

**Table 2:** Corrected RGB values (ΔRGB) and associated standard deviations for CGA detection using AgFAU

CGA (mg L^−1^)	ΔR ± SD	ΔG ± SD	ΔB ± SD
1.0	9.0 ± 1.2	6.8 ± 1.5	0.9 ± 5.0
2.5	−0.7 ± 2.3	−8.2 ± 1.9	−11.5 ± 2.7
5.0	−25.9 ± 1.6	−34.7 ± 1.5	−37.0 ± 2.3
7.5	−26.8 ± 2.7	−37.1 ± 2.7	−36.7 ± 2.3
10.0	−29.2 ± 2.6	−39.3 ± 2.3	−41.0 ± 2.6
15.0	−34.0 ± 1.7	−44.9 ± 0.9	−44.0 ± 2.5
20.0	−45.9 ± 2.8	−52.6 ± 2.9	−43.4 ± 3.6

The AgFAU colorimetric response toward CGA was evaluated by fitting the individual RGB parameters using polynomial regression analysis. One of the main advantages of RGB analysis is the ability to correlate changes in light intensity within specific wavelength regions to variations in color, enabling quantitative interpretation of colorimetric responses (Xing et al., 2020). In addition, RGB-based models have been reported to provide good correlation and reliable analytical performance when compared with other color space approaches, such as value (V), saturation (S), and grayscale models (Xing et al., 2022).

The corrected signals (ΔR, ΔG, and ΔB) showed a non-linear dependence on CGA concentration over the studied range. Therefore, cubic polynomial models were used to describe the analytical response. The obtained fits showed good agreement with the experimental data, with determination coefficients (R^2^) of 0.9819, 0.9844, and 0.9248 for ΔR, ΔG, and ΔB, respectively. Residual analysis showed a random distribution around zero, indicating that the selected model adequately describes the experimental data. The non-linear RGB response may be attributed to combined light absorption and scattering effects in the AgFAU suspension, resulting in a concentration-dependent behavior that is better described by polynomial models.

Although the cubic model adequately represents the overall behavior, a linear range at lower concentrations (1–10 mg L^−1^) was selected for analytical purposes. The LOD and LOQ values for each RGB parameter were calculated based on this linear region. As shown in Table 3, the LOD values obtained for R (0.83 mg L^−1^), G (0.47 mg L^−1^), and B (1.17 mg L^−1^) resulted in an average value of 0.8 ± 0.3 mg L^−1^. The average LOQ was 2.7 ± 1.2 mg L^−1^, which is close to the lowest concentration level evaluated, indicating the adequacy of the method for CGA detection (Fig. 5).

**Table 3:** Linear calibration parameters for CGA determination based on RGB signals (1–10 mg L^−1^)

	Channel	Linear equation	*R*^2^	LOD (mg L^−1^)	LOQ (mg L^−1^)
RGB color absorbance	R	*y* = −4.363*x* + 7.966	0.825	0.83	2.75
	G	*y* = −5.130*x* + 4.178	0.823	0.47	1.56
	B	*y* = −4.626*x* − 1.007	0.821	1.17	3.89

The limits of detection obtained in the present work (0.47–1.17 mg L^−1^, corresponding to 1.3–3.3 µmol L^−1^) are comparable to values reported in the literature for CGA determination using colorimetric approaches (Table 3). The colorimetric response of the AgFAU system is associated with the interaction between CGA and silver species immobilized within the zeolite framework, resulting in concentration-dependent color variations.

As summarized in Table 4, the AgFAU-based system exhibits analytical performance consistent with other reported colorimetric platforms for CGA detection. The main advantages of AgFAU include its structural stability and good dispersion in aqueous media, which represent favorable characteristics for reliable and practical CGA monitoring.

**Table 4:** Colorimetric sensors for chlorogenic acid (CGA) detection

Sensor system	Range (µmol L^−1^)	LOD (µmol L^−1^)	Reference
AuNPs (red)	3–25	8.7	Scroccarello et al. (2019)
NaIO_4_/3-methyl-2-benzothiazolinone hydrazine (MBTH) (pink color)	70–710	2	Hidayat et al. (2017)
Tetramethyl benzidine (TMB) radical (blue color) l by TMB/H_2_O_2_ using tantalum (Ta) metal–organic framework	0–20	0.11	Karami et al. (2020)
Fe_4_[Fe(CN)_6_]_3_ Prussian blue by FeCl_3_/K_3_Fe(CN)_6_ reaction	0–56	2.8	Tao et al. (2023)
Ag-FAU in buffer medium (pH 10.8)	0–56	0.8	This work

With respect to the reproducibility of the analytical measurements, a high degree of homogeneity was observed for CGA samples prepared at a concentration of 10 mg L^−1^ and analyzed in quintuplicate (*n* = 5). The relative standard deviation (RSD) values obtained for the RGB parameters were 3.9%, 3.0%, and 5.3% for ΔR, ΔG, and ΔB, respectively. These results indicate good repeatability of the AgFAU colorimetric response for CGA detection, with low RSD values for all RGB parameters.

#### Effect of potential interferents

**Fig. 6:**
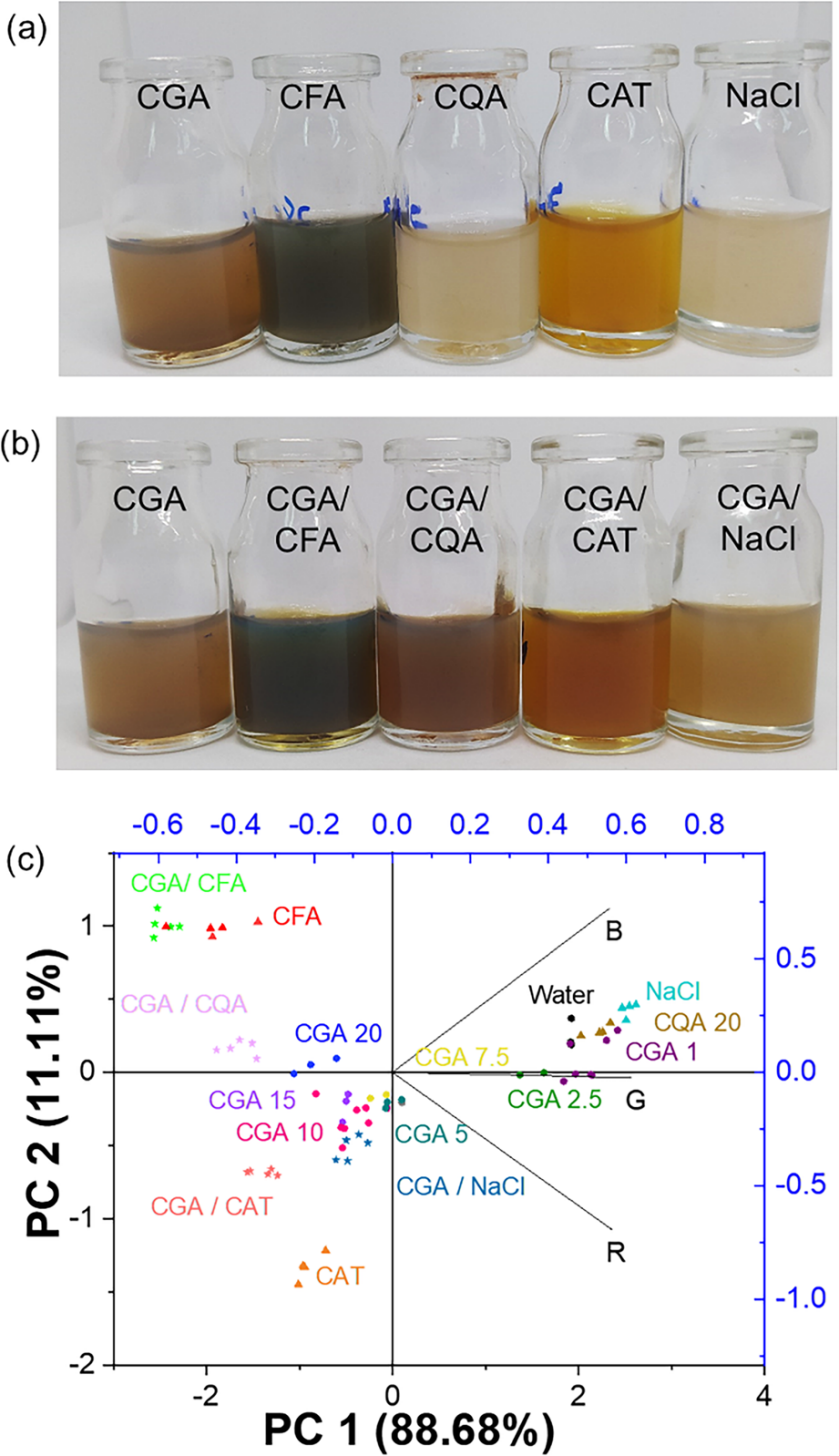
Colorimetric response of CGA compared with pure compounds (**a**) and CGA in the presence of potential interferents (**b**) and principal component analysis (PCA) of the corresponding RGB color parameters (**c**)

The effect of potential interferents on CGA detection was evaluated using caffeic acid (CFA), quinic acid (QA), catechin (CAT), and a sodium chloride solution (0.05%, w/v). Figure 6a shows that the colorimetric responses of the individual interferent solutions differ from that observed for CGA at the same concentration (20 mg L^−1^), indicating the absence of false-positive responses under the investigated conditions.

As shown in Fig. 6b, when CGA is present together with the interferents, CFA exhibits the most pronounced visual interference, partially masking the CGA response due to its intense gray-green coloration at high concentration. In contrast, QA and CAT display color responses that remain distinguishable from CGA. Under alkaline conditions, the polyphenol solutions exhibit different visual appearances, ranging from pale yellow to greenish tones, which influence the resulting colorimetric signal.

Principal component analysis (PCA) of the RGB color parameters (Fig. 6c) further supports these observations, showing that the evaluated interferents occupy distinct regions in the multivariate space relative to CGA, thus reducing the likelihood of false-positive identification. QA and NaCl cluster closer to the blank and to CGA at low concentration (1 mg L^−1^), which represents the practical monitoring limit in this study. Upon addition of interferents to CGA at 20 mg L^−1^, a shift in the PCA scores is observed in all cases. Notably, the CGA/QA system exhibits partial overlap with the QA region, consistent with their similar visual responses at high concentration.

#### CGA monitoring in coffee washing water

**Fig. 7:**
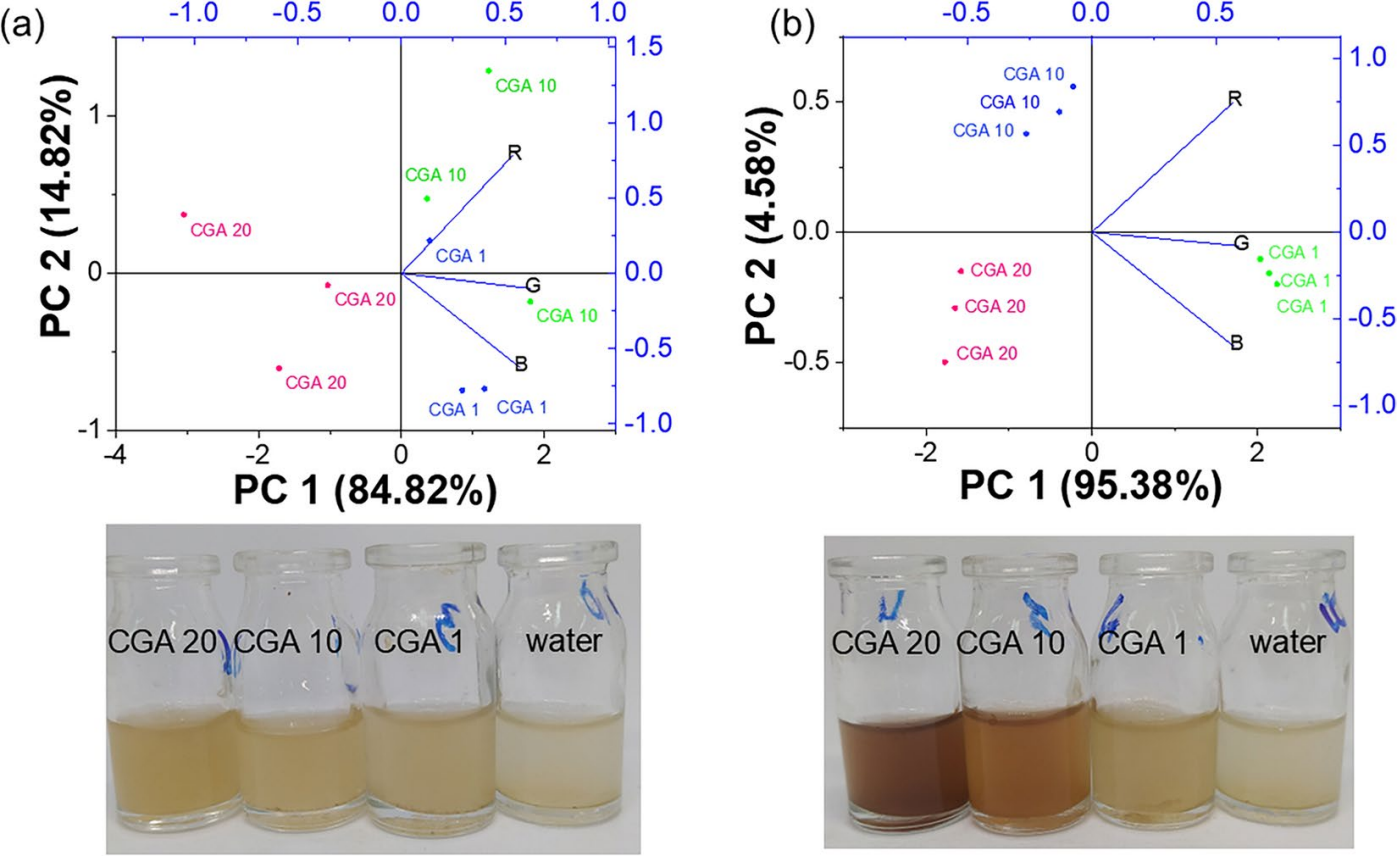
CGA monitoring present in washing water of coffee grain with dilutions of 10× (**a**) and 50× (**b**)

CGA monitoring was evaluated using washing water obtained from dried coffee beans after washing, centrifugation, and dilution of the samples in order to minimize the intense yellowish coloration of the matrix. Among the tested dilution factors (10× and 50×), only the 50× dilution provided a clear and distinguishable colorimetric response across different CGA concentrations, as shown in Fig. 7 (Table S3). Considering that dried coffee beans were employed, the native CGA concentration in the washing water would be minimal. Thus, the experiments focused on the analysis of CGA-added samples to assess the influence of the coffee matrix on the colorimetric response.

This behavior may be attributed to the presence of residual compounds in the 10× -diluted sample, which, despite appearing visually clear, can still interfere with the colorimetric response of AgFAU toward CGA. Consistently, PCA analysis revealed a higher dispersion of replicate points for the 10× dilution, indicating reduced homogeneity of the analytical response. In contrast, the 50× -diluted samples exhibited improved separation between CGA concentrations and greater reproducibility among replicates. These results indicate that appropriate dilution of coffee washing water is required to ensure reliable CGA monitoring using the AgFAU colorimetric system.

Based on these observations, the 50× dilution was selected for further evaluation of the analytical performance in the coffee matrix. This dilution was selected to minimize matrix background while preserving analytical sensitivity. Recovery experiments were then carried out using the diluted washing water in order to assess the applicability of the proposed method under real sample conditions. Known amounts of CGA were added to the 50× -diluted matrix to obtain final concentrations of 10 and 20 mg L^−1^.

The concentration found (*C*_found_) was calculated from the linear calibration equations obtained for each RGB channel. The corresponding recovery values, expressed as the ratio between the found and added concentrations, are summarized in Table 5.

**Table 5:** Recovery values for CGA determined in 50 × -diluted coffee wastewater using RGB-based colorimetric analysis

Channel	*C*_added_ (mg L^−1^)	*Δ* signal	*C*_found_ (mg L^−1^)	Recovery (%)
R	10.0	−56	14.66	146.6
R	20.0	−100	24.75	123.8
G	10.0	−82	16.80	168.0
G	20.0	−105	21.28	106.4
B	10.0	−59	12.53	125.3
B	20.0	−62	13.18	65.9

As observed, the recovery values depend on both the concentration level and the selected RGB parameter. At 20 mg L^−1^, the green channel (ΔG) provided the most accurate result, with a recovery of 106.4%, indicating good agreement with the expected value. In contrast, higher deviations were observed at 10 mg L^−1^ for all channels, suggesting the influence of matrix effects at lower concentration levels. These results indicate that the proposed method is suitable for CGA detection in diluted coffee washing water, particularly at higher concentration levels, where better agreement with the expected values was observed.

#### Proposed mechanism of colorimetric response

**Fig. 8:**
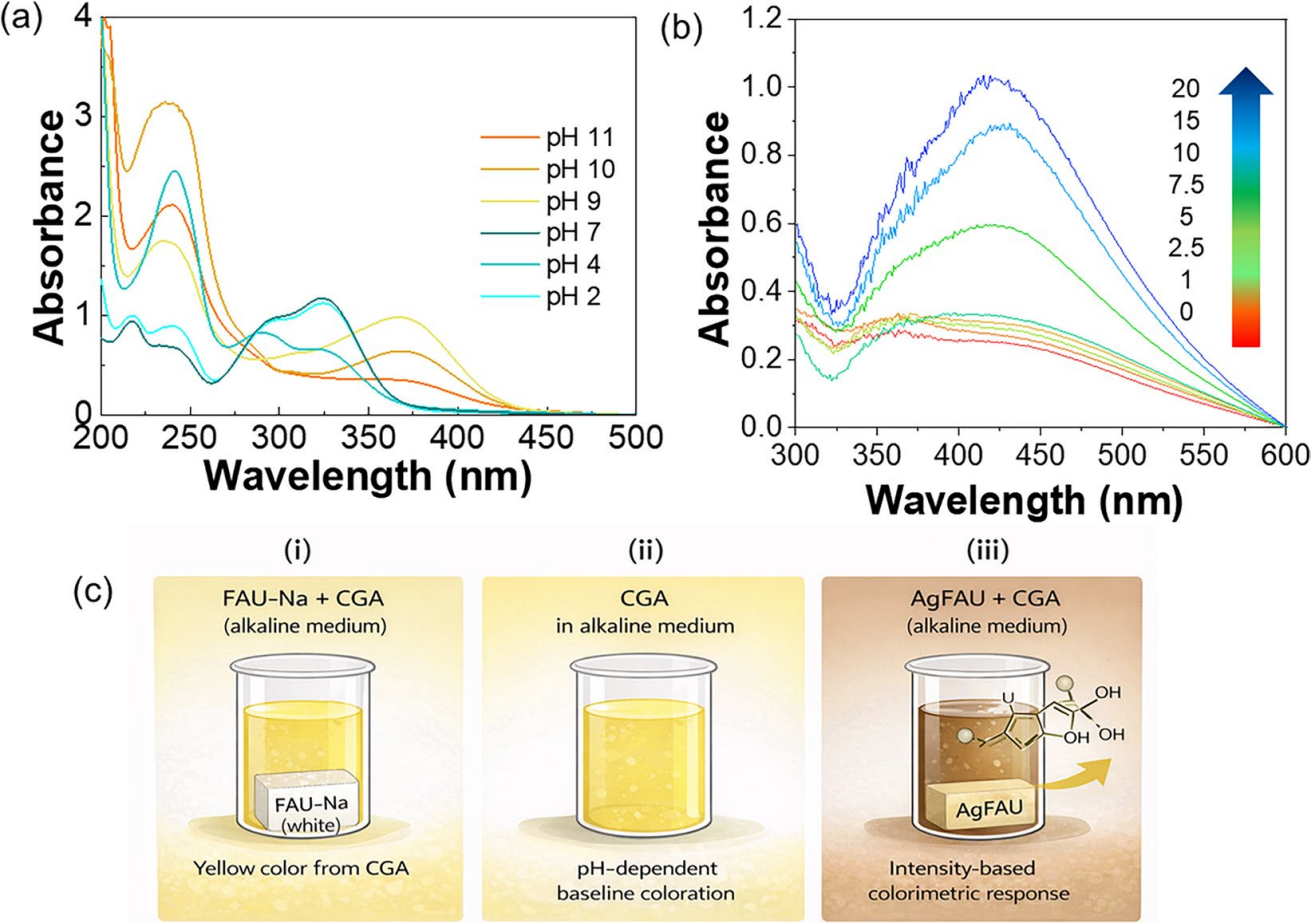
**a** UV-vis spectra of CGA at different pH values. **b** UV-vis spectra of AgFAU dispersions in alkaline medium in the presence of CGA, and **c** schematic representation of the proposed intensity-based colorimetric response of the AgFAU sensor toward CGA under alkaline conditions

The molecular structure of chlorogenic acid (CGA) is strongly influenced by the pH of the solution, as evidenced by changes in its UV-vis absorption profile (Fig. 8a). At pH values above 7, the appearance of a broad absorption band around 400 nm is observed, accompanied by the development of a yellowish coloration. According to the literature, CGA undergoes pH-induced structural transformations in alkaline media, including the formation of quinone-type species, as well as changes in the electronic distribution of phenolic groups that affect its optical properties (Namazian & Zare, 2005).

Figure 8b shows the UV-vis spectra of Ag-modified faujasite (AgFAU) dispersions in alkaline medium. Even in the absence of CGA, the zeolite dispersion exhibits a broad background absorption in the UV-vis region, indicating an intrinsic optical response under basic conditions. Upon CGA addition, no new absorption bands or significant spectral shifts are detected. Instead, increasing CGA concentration mainly results in an enhancement of the overall absorption intensity. This behavior indicates that CGA detection does not involve the formation of new UV-vis active chromophoric species, but rather a modulation of the intrinsic optical response of the AgFAU dispersion, consistent with an intensity-based colorimetric mechanism.

The role of silver species was further elucidated by comparison with the sodium-exchanged zeolite (NaFAU). NaFAU remained optically inactive, exhibiting a white appearance in aqueous suspension. In alkaline medium, NaFAU preserved the pale-yellow coloration of CGA with only a slight increase in turbidity, confirming that the baseline color originates from CGA itself. In contrast, AgFAU produced a markedly stronger yellow-to-brown color response, highlighting the central role of Ag-containing sites in amplifying the optical signal. Since the FAU crystalline framework is preserved after ion exchange, this enhancement can be attributed to the presence of silver species rather than to structural differences in the zeolite support.

In turbid dispersions, perceived color is governed not only by molecular absorption but also by light scattering effects associated with suspended particles. Under alkaline conditions, AgFAU presents an intrinsic background absorption in the visible region, which is progressively intensified upon CGA addition due to combined absorption and scattering phenomena. As a result, a gradual yellow-to-brown color transition is observed without the emergence of new absorption features or spectral shifts. Similar intensity-based colorimetric responses have been reported for sensor systems in which signal amplification arises from optical attenuation and scattering rather than from the generation of new chromophores (Piriya et al., 2017; Lan et al., 2019; Jia et al., 2021).

The proposed mechanism, schematically illustrated in Fig. 8c, describes an intensity-based colorimetric response of AgFAU toward CGA under alkaline conditions. Although redox interactions between CGA and silver species have been reported in other systems (Abdalameer et al., 2021; Elsayed et al., 2021; Masum et al., 2019; Tošović et al., 2017; Zhang et al., 2016), the present UV-vis results do not provide spectroscopic evidence for metallic silver formation or radical-mediated processes. Instead, CGA selectively amplifies the intrinsic optical response of the silver-modified zeolite dispersion. Similar intensity-based colorimetric responses have been reported for sensor systems in which signal amplification arises from optical scattering and attenuation rather than from the formation of new chromophoric species, particularly in nanoparticle- and metal-based colorimetric sensors, where aggregation, dispersion, and light interference effects dominate the visual response (Piriya V.S et al., 2017).

**Fig. 9:**
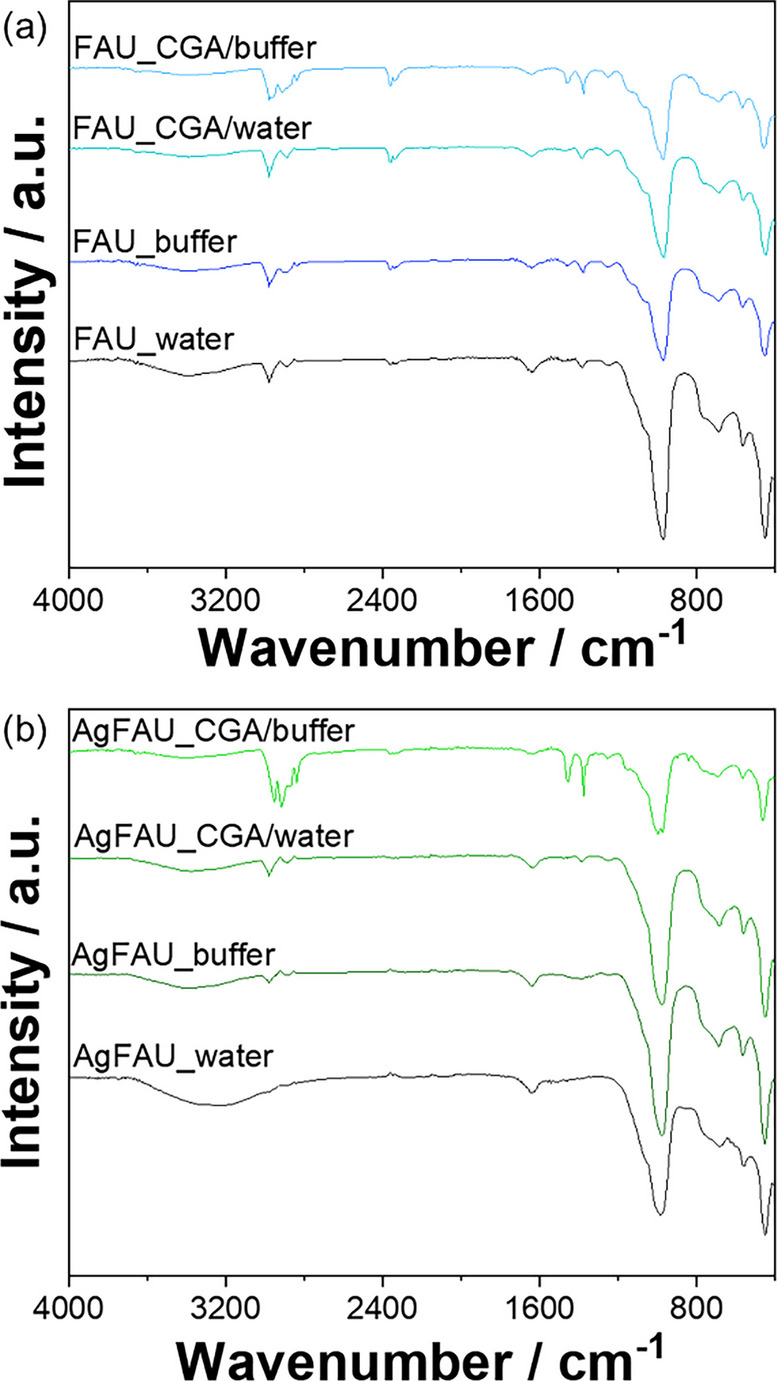
FTIR spectra of FAU (**a**) and AgFAU (**b**) after contact with water, buffer solution, and chlorogenic acid (CGA) under different conditions

In Fig. 9, the FTIR analysis showed more noticeable changes after contact with CGA in a buffered medium, especially in the regions around 3000–3600 cm^−1^ and 1500–1700 cm^−1^. These variations may be associated with contributions from O-H groups and carbonyl or aromatic structures of CGA-derived species interacting with the zeolite surface. Similar changes were also observed for the non-modified FAU, suggesting that the zeolite itself may already interact with the analyte. However, the superior colorimetric response observed for AgFAU suggests that silver may play an important role in enhancing the optical signal. Therefore, the sensing mechanism is likely related to surface interactions that are enhanced in the presence of silver, rather than the formation of a new chromophore.

Interference studies further support this mechanism. As shown in Fig. 6b, different polyphenols exhibited distinct visual responses in alkaline medium. While CGA and catechin produce pale yellow tones, caffeic acid induces a gray-green coloration that can partially mask the CGA signal. The NaFAU control remains optically inactive, confirming that the enhanced colorimetric response observed for AgFAU arises from the interaction between CGA and silver-containing sites rather than from the zeolite matrix itself. Overall, the colorimetric response results from the combination of pH-dependent optical properties of polyphenols and their interaction-induced modulation of the AgFAU dispersion.

## Conclusions

A silver-modified faujasite zeolite (AgFAU) was developed as a colorimetric platform for chlorogenic acid (CGA) monitoring, exhibiting a clear concentration-dependent response quantified by smartphone-assisted RGB analysis. The system operated in the 1–20 mg L^−1^ concentration range and achieved a limit of detection in the range of 0.47–1.17 mg L^−1^, with good repeatability (RSD ≤ 6%), reinforcing its reliability for practical applications. The applicability of the method was demonstrated using coffee washing water as a representative matrix, where appropriate sample dilution (50 ×) enabled reliable monitoring of added CGA while minimizing matrix effects. Overall, the AgFAU platform represents a simple and portable alternative for CGA monitoring, with potential for quality control applications and future mechanistic investigations.

## Supplementary information

Below is the link to the electronic supplementary material.

ESM 1 (DOCX 76.3 KB)

## Data Availability

The data that support the findings of this study are available from the corresponding author upon reasonable request.
