# Supplementary material for: Simple colorimetric monitoring of chlorogenic acid in coffee washing water using Ag-modified faujasite zeolite
Source: Environ Monit Assess. 2026 May 13;198(6):582. doi: 10.1007/s10661-026-15430-x (PMC13171940; doi:10.1007/s10661-026-15430-x)

**Table S1.** RGB color space parameters of curve calibration were obtained by smartphone app image (RGB color detector).


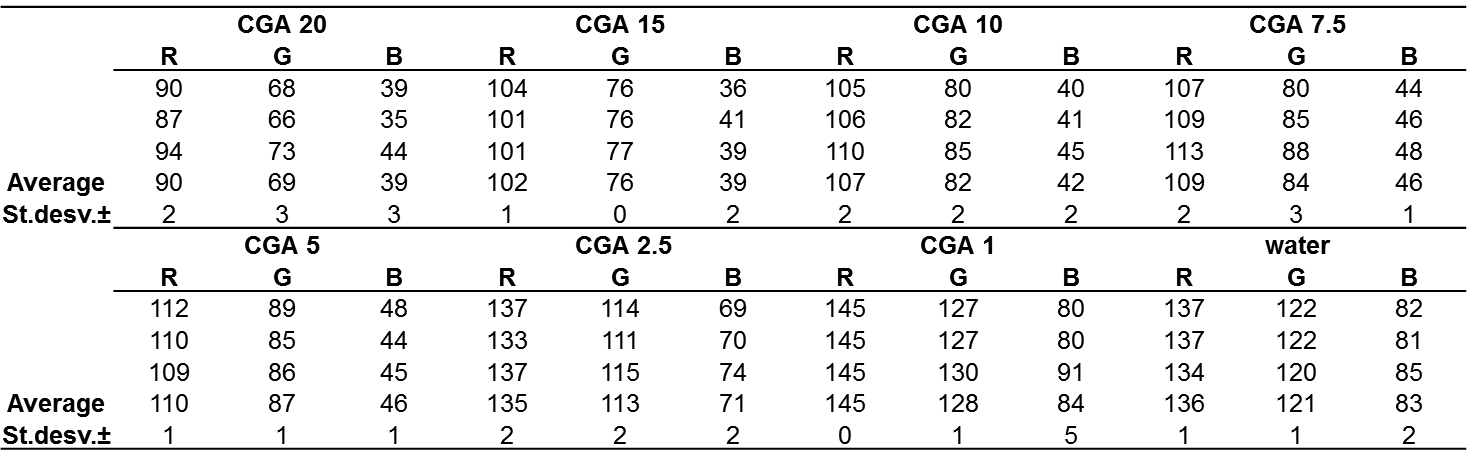


**Table S2.** RGB color space parameters of analyte interferents obtained by smartphone app image (RGB color detector).


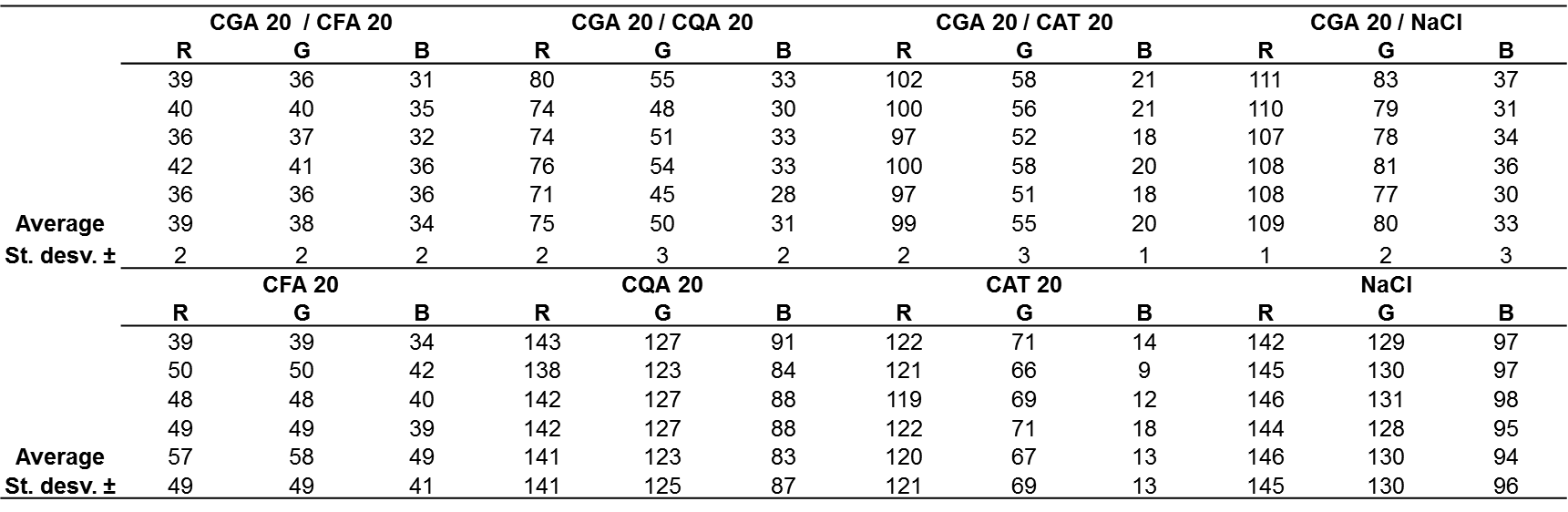


**Table S3.** RGB color space parameters of CGA in coffee water diluted (10x and 50x) obtained by smartphone app image (RGB color detector).


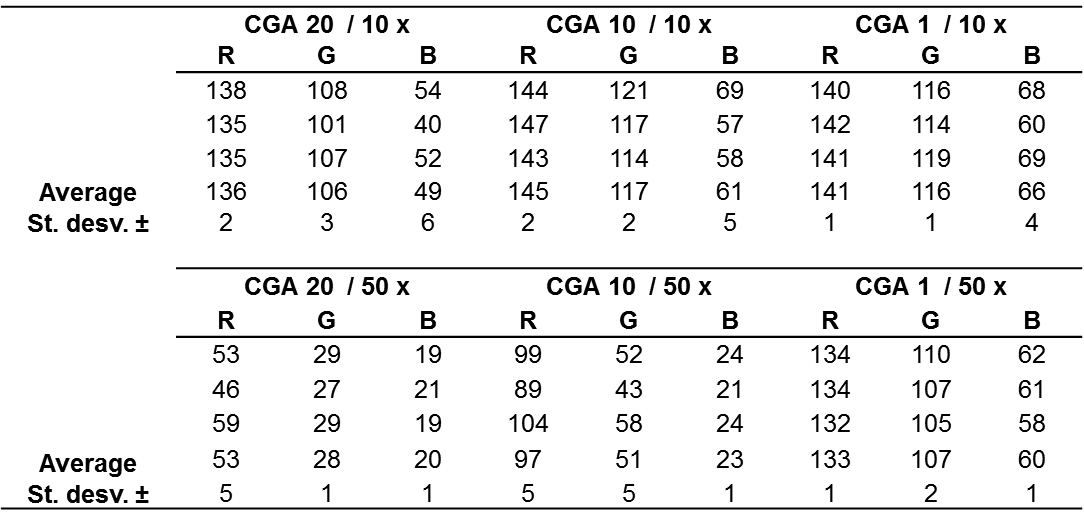

Supplement: Supplementary file 1 — (DOCX 76.3 KB) [file 10661_2026_15430_MOESM1_ESM.docx]
